# P-216. US Health Care Spending on Septicemia

**DOI:** 10.1093/ofid/ofaf695.438

**Published:** 2026-01-11

**Authors:** Yan Tian, Wenjun Wang

**Affiliations:** Second Hospital of Xi'an Jiaotong University, Xi'an, Shaanxi, China; Second Hospital of Xi'an Jiaotong University, Xi'an, Shaanxi, China

## Abstract

**Background:**

Septicemia imposes a substantial and growing burden on the US health care system. This study aims to estimate health care spending on septicemia from 2010 to 2019, examining variation by sex, age, type of care, and geographic location to inform policy and resource allocation.

**Figure:**
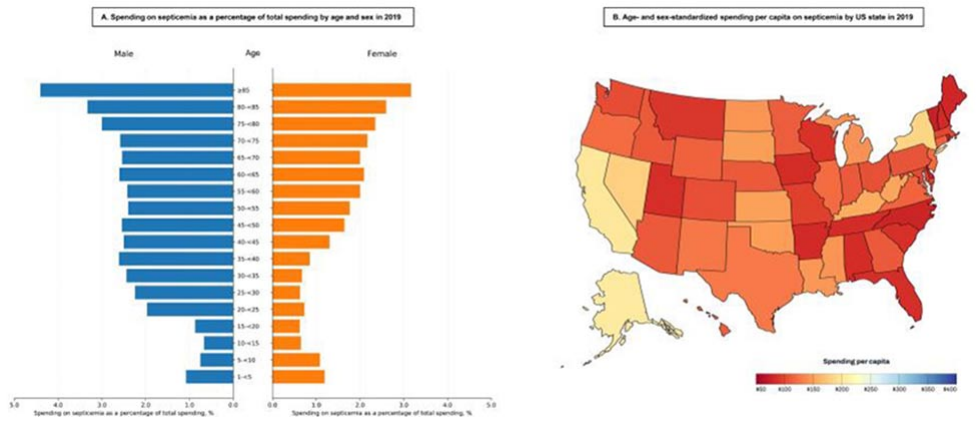

**Methods:**

Using data from the US Health Care Spending by Condition database, we analyzed inflation-adjusted (2019 USD) spending estimates for septicemia. Spendings were stratified by sex, age groups, care categories, and state-level distribution.

**Results:**

Health care spending on septicemia increased from $34 billion in 2010 (95% CI, $32 billion–$35 billion) to $51 billion (95% CI, $49 billion–$53 billion) in 2019. The proportion of health care spending attributed to septicemia increased from 1.67% (95% CI, 1.59%–1.72%) in 2010 to 2.14% (95% CI, 2.05%–2.24%) in 2019. In 2019, males accounted for $28 billion (95% CI, $27 billion–$29 billion), while female accounted for $23 billion (95% CI, $22 billion–$24 billion). By age group, individuals aged ≥65 years had the highest spending ($27 billion; 95% CI, $26 billion–$29 billion), followed by those aged 45–64 years ($15 billion; 95% CI, $14 billion–$16 billion), 20–44 years ($7.2 billion; 95% CI, $6.7 billion–$7.6 billion), and < 20 years ($2.1 billion; 95% CI, $1.9 billion –$2.3 billion). Hospital inpatient care constituted the largest share of spending (94.1%), with nursing facility care, ambulatory care, home health care, emergency department care, and prescribed retail pharmaceuticals accounting for the remainder. At the state level, age- and sex-standardized spending per capita ranged from $218 (95% CI, $189–$261) in Alaska, to $99 (95% CI, $87–$116) in Vermont.

**Conclusion:**

Health care spending on septicemia has increased significantly from 2010 to 2019, with substantial variation by sex, age, type of care, and geographic location. These findings highlight the growing economic burden of septicemia and underscore the need for targeted interventions to reduce disparities and improve the efficiency of care delivery.

**Disclosures:**

All Authors: No reported disclosures

